# Supplementary material for: Chromothripsis during telomere crisis is independent of NHEJ, and consistent with a replicative origin
Source: Genome Res. 2019 May;29(5):737–49. doi: 10.1101/gr.240705.118 (PMC6499312; doi:10.1101/gr.240705.118)
Supplement: Supplemental Material [file supp_gr.240705.118_Supplemental_file_1.zip › contigs/annotated_contigs/DB78/contig.2.DB78_length_560_mean_cov_6.8.docx]

**DB78_length_560_mean_cov_6.8**

```
ATGACGCATTAGCCAAAAAACTTTGAATTTTTTATACTAGTCTATTTCGGCCCAGAACCACCACAGAAAATTGCC|AAAAAAAAACAAA
                                                                            >chr2:785512
CATGATATGAACAGGCTCTCATAAGTCCACAGTTTCTGACATGAGAAAAATAAAAACAATAACACAGGAAGTACGTTGAACATCAAAGT
38-78551484 - E=2e-133
TACTATTAGCTTCCAATCTGACCATTTCAGCATCACTCATTCAAAGGGAGCTGATGATTTGAAAGAAAAATATAGTTTCATTGTTTGTA

TTTTTAATTGAGATATAATTGACAAAATAAACTCCACCTATTTAAAATATATTT|T|AGTATCTTGATTACTGTAACTTTATAATAATG
                                                       >chr2:78548925-78549165 - E=9e-1
ATAAAACTCAGGTAATATTAGCCCTGAAAATTTGTCCTATTTTTAAAATTTTACTTTAAGTTCTGGGATACAAGTGCGGAACGTGCAGG
33
TTTGTTACATGAGTATACATATGCCATAGTTTACTGCACCTATCAACCCATCATCAAGGTTTTAAGATCCACATGCATTAGTTATTTGT

CCTAATGTTTTCCCTCCCCTCATCTCCCA
```
